# Development of a risk model for low oocyte retrieval in first-cycle IVF patients with diminished ovarian reserve: a retrospective single-center study

**DOI:** 10.1530/RAF-25-0120

**Published:** 2026-01-13

**Authors:** Jin Lu, Chenyue Dong, Lu Wang, Peizhe Tian, Cuilian Zhang, Xue Wang

**Affiliations:** ^1^ Department of Reproductive Medicine Center, Henan Provincial People’s Hospital, Zhengzhou, People’s Republic of China; ^2^ Department of Reproductive Medicine Center, Zhengzhou University People’s Hospital, Zhengzhou, People’s Republic of China

**Keywords:** low oocyte retrieval, diminished ovarian reserve, trigger-to-retrieval time, nomogram

## Abstract

**Abstract:**

Low oocyte retrieval (LOR), defined as oocytes retrieved from <50% of mature follicles, significantly impacts IVF success, especially in patients with diminished ovarian reserve (DOR). Predictive models tailored for this population are limited. This retrospective cohort study developed a predictive nomogram for LOR risk in first-cycle IVF/ICSI patients with DOR. The data from 2,594 eligible patients were analyzed. LOR was defined as oocyte retrieval rate <50% (oocytes/follicles ≥14 mm on trigger day). Least absolute shrinkage and selection operator (LASSO) regression identified predictors from clinical/endocrine parameters. A multivariate logistic model was built, visualized as a nomogram, and internally validated (bootstrap resampling). Performance was assessed via AUC, calibration, and decision curve analysis. Eight independent predictors were identified: age, basal FSH, AFC, AMH, LH on hCG day, progesterone (P) on hCG day, number of large follicles (≥14 mm) on hCG day, and trigger-to-retrieval time (TR-OPU). The model demonstrated moderate yet acceptable discrimination (AUC = 0.721, 95% CI: 0.678–0.772) and calibration. TR-OPU was significantly shorter in the LOR group (34.85 vs 35.05 h, *P* < 0.001). DCA confirmed clinical utility. This study establishes a clinical-endocrine nomogram predicting LOR risk in first-cycle DOR patients. Incorporating key factors, such as TR-OPU, ovarian reserve markers (AMH, AFC, and bFSH), and trigger-day hormones, it may help stratify risk but requires external validation before clinical application.

**Lay summary:**

Women with naturally lower egg reserves often face extra challenges with IVF. A common problem is retrieving fewer eggs than expected during the procedure (‘low oocyte retrieval’ or LOR), which significantly lowers the chance of pregnancy. Doctors lack good tools to predict who is most at risk. Our study analyzed data from 2,594 women with low egg reserves undergoing their first IVF cycle. We created a simple prediction chart that combines key information easily available to doctors: the woman’s age, standard hormone blood tests (AMH and FSH), an ultrasound count of egg sacs (follicles), hormone levels on the day of the final IVF trigger shot, and critically – the exact number of hours between the trigger and the egg collection surgery. This chart accurately estimates an individual woman’s risk of LOR. Knowing this risk beforehand helps doctors personalize treatment timing and medication, aiming to collect more eggs and improve the chances of a successful pregnancy, while helping patients avoid unnecessary emotional and financial strain.

## Introduction

The success of *in vitro* fertilization (IVF), a key treatment for infertility, largely depends on retrieving a sufficient number of high-quality oocytes. However, low oocyte retrieval (LOR) – defined as retrieval of oocytes from <50% of follicles ≥14 mm (Paffoni *et al.* 2022, Lin *et al.* 2024) – is particularly prevalent in certain clinical contexts, especially among patients with diminished ovarian reserve (DOR). While the absolute number of oocytes is a crucial determinant of IVF success, the retrieval rate provides a distinct and critical measure of cycle efficiency that is particularly salient for DOR patients. In this population, characterized by a low baseline number of follicles, a suboptimal retrieval rate indicates a profound loss of potential and points to inefficiencies in the final stages of follicular maturation or the retrieval process itself, beyond the expected challenge of low numbers. LOR not only compromises treatment efficacy but also contributes to greater psychological stress and financial burden. Therefore, accurately predicting this risk and implementing timely interventions are crucial to improving overall clinical outcomes.

Numerous studies have identified several factors influencing oocyte retrieval rates, including age, body mass index (BMI), ovarian reserve, and stimulation protocols (Frattarelli & Kodama 2004, Devesa *et al.* 2018, Yan *et al.* 2022, Wang *et al.* 2023), all of which may impact the asynchronous maturation of follicles. One critical factor is the interval from trigger injection to oocyte retrieval, known as trigger-to-retrieval time (TR-OPU). Prolonging the TR-OPU to more than 35 h has been shown to significantly reduce the risk of LOR, particularly in younger patients and those with established ovarian insufficiency (Deng *et al.* 2020, Shen *et al.* 2020). Optimizing this interval not only increases the oocyte yield but also enhances pregnancy outcomes.

While several models, including our previous work (Xu *et al.* 2024), primarily forecast a low absolute oocyte yield, which is useful for identifying ‘poor responders’ (Scheinhardt *et al.* 2018). For patients with DOR who inherently face a risk of low yield, predicting cycle efficiency – defined as the oocyte retrieval rate – is a distinct and critical clinical question. In addition, conditions, such as endometriosis, can lead to follicular fibrosis and increased viscosity of follicular fluid, which ultimately affect the oocyte retrieval rate (Sanchez *et al.* 2017). Optimizing follicular flushing can enhance oocyte retrieval (Georgiou *et al.* 2022).

Although several studies have examined various factors influencing oocyte retrieval, individualized and cycle-specific predictive models for patients with DOR remain limited. Effective predictive tools can assist clinicians in identifying high-risk patients early in the stimulation cycle, thereby facilitating more rational and personalized clinical decision-making. Such tools may not only alleviate psychological stress but also enhance the cost-effectiveness of IVF and improve the overall patient experience and outcomes.

This study aims to develop a predictive model for low oocyte yield in DOR patients. Using existing clinical data, we seek to construct an intuitive and user-friendly nomogram by quantifying the association between selected variables and the risk of LOR. This model is intended to enable clinicians to assess individual patient risk more accurately in routine practice. Ultimately, we aim to provide a practical tool for the personalized management of DOR patients and contribute to the continued advancement and refinement of assisted reproductive technologies.

## Materials and methods

### Study design

This retrospective cohort study aimed to develop a model for predicting the risk of low oocyte retrieval LOR in women with DOR. The study population consisted of 24,450 patients who underwent IVF or intracytoplasmic sperm injection (ICSI) at the Reproductive Center of Henan Provincial People’s Hospital between January 2017 and June 2023. All patients undergoing their first IVF cycle and meeting the diagnostic criteria for DOR were included. The diagnostic criteria for DOR were as follows: i) anti-Müllerian hormone (AMH) < 1.1 ng/mL; ii) basal follicle-stimulating hormone (bFSH) ≥ 10 IU/L (Li *et al.* 2023); and iii) antral follicle count (AFC) < 7, with at least two of the three criteria required for diagnosis (Expert Consensus Group on Diminished Ovarian Reserve 2022). Exclusion criteria included: i) cycles using donor oocytes, ii) 110 cycles that were canceled before the oocyte retrieval procedure, iii) cycles with no follicles ≥14 mm in diameter on the day of human chorionic gonadotropin (HCG) administration, and iv) cycles with luteinizing hormone (LH) levels >10 IU/mL on the HCG day. A total of 2,594 patients met the inclusion criteria and were enrolled in the study. LOR was defined as an oocyte retrieval rate of less than 50%, calculated as the ratio of retrieved oocytes to the number of mature follicles (≥14 mm in diameter on ultrasound).

This retrospective study was conducted in accordance with the Declaration of Helsinki and was approved by the Reproductive Medicine Ethics Committee of Henan Provincial People’s Hospital committees (Approval No: SYSZ-LL-2021091501). The requirement for written informed consent was waived by the ethics committee due to the retrospective design and the use of de-identified data. All patient data were anonymized before analysis to ensure confidentiality.

### IVF program

The protocols for ovarian stimulation, oocyte retrieval, embryo culture, morphological grading, vitrification, cryopreservation, and subsequent thawed embryo transfer have been previously described (Chen *et al.* 2015). Oocytes were fertilized using either conventional IVF or ICSI. Ovarian stimulation was routinely performed using one of the following protocols: gonadotropin-releasing hormone agonist (GnRHa), GnRH antagonist (GnRH-ant), microstimulation, or progesterone-primed ovarian stimulation (PPOS). Trigger timing was determined based on the diameter and number of dominant follicles, duration of gonadotropin administration, and serum hormone levels. Final oocyte maturation was induced using 5,000–10,000 IU human chorionic gonadotropin (hCG; Chorionic Gonadotrophin; Shanghai, China), 0.2 mg GnRHa (ganirelix (Orgalutran; NV Organon, The Netherlands) or cetrorelix (Cetrotide; Merck Serono Europe Ltd, UK)), or a combination of hCG and GnRHa. The decision to administer the trigger was based on expert consensus criteria: either three dominant follicles ≥17 mm or two dominant follicles ≥18 mm in diameter, combined with changes in serum estradiol and progesterone levels (Qiao *et al.* 2015). Oocyte retrieval was scheduled approximately 34–36 h after triggering. The timing was adjusted as necessary according to follicular development and hormone profiles on the day of hCG administration and the following day. The choice between conventional IVF and ICSI was based on semen parameters. Embryos were cultured *in vitro* to day 3 or day 5, and high-quality embryos were selected for fresh transfer. Embryo quality was evaluated by a single embryologist using established morphological criteria (Gardner *et al.* 2000, Alpha Scientists in Reproductive Medicine and ESHRE Special Interest Group of Embryology 2011).

### Statistical analysis

The primary outcome of this study was the probability of low oocyte yield in patients with DOR. Statistical analyses were performed using the EmpowerStats software (version x.x, X&Y Solutions Inc., USA). Continuous variables with non-normal distributions were summarized as medians and interquartile ranges. Comparisons between groups were conducted using the chi-square test or Fisher’s exact test for categorical variables, and Student’s *t*-test or Wilcoxon rank-sum test for continuous variables, as appropriate.

To identify independent predictors and develop an individualized predictive model for low oocyte yield, a least absolute shrinkage and selection operator (LASSO) logistic regression was employed. Candidate predictors included TR-OPU along with other clinical features. Variable selection and model fitting were performed using the ‘glmnet’ package in R, which implements efficient regularization techniques combining LASSO and ridge regression to enhance model performance and prevent overfitting. A nomogram was subsequently constructed using the ‘rms’ package to provide a visual tool for estimating individual risk.

Model robustness was internally validated via bootstrap resampling with 1,000 iterations to minimize optimism bias and yield reliable performance estimates. For each bootstrap sample, model parameters were re-estimated, and a shrinkage factor was calculated to adjust the final model coefficients accordingly. The predictive accuracy of the nomogram was assessed by the area under the receiver operating characteristic curve (AUC), with values ranging from 0.5 (no discrimination) to 1.0 (perfect discrimination). Calibration was evaluated graphically through calibration plots to assess agreement between the predicted and observed outcomes. Furthermore, decision curve analysis (DCA) was conducted to quantify the net clinical benefit across a range of threshold probabilities.

The sample size adequacy for this prediction model was assessed post-hoc according to contemporary methodological standards (Riley *et al.* 2020). With 113 observed events of LOR and eight predictor parameters in the final model, our study achieves an event-per-variable (EPV) ratio of 14.1. This exceeds the recommended minimum of 10 EPV for developing logistic prediction models, thereby mitigating the risk of overfitting and ensuring stable and reliable parameter estimates.

All statistical tests were two-sided, with *P* values < 0.05 considered statistically significant.

## Results

### Patient characteristics

A total of 2,594 patients with a mean age of 36.6 ± 5.7 years were included in this study. Among them, 113 cases (4.36%) were classified into the LOR rate group. No significant differences were observed between groups in terms of age, BMI, duration of infertility, or infertility type (*P* > 0.05). The basal LH was higher in the LOR group (5.30 vs 4.80, *P* = 0.026). The AMH levels (0.48 vs 0.68, *P* < 0.001) and AFC (2.93 vs 4.05, *P* < 0.001) were significantly higher in the high oocyte retrieval group. The distribution of ovarian stimulation protocols differed significantly between the two groups (*P* < 0.001). Statistically significant differences were also observed in estradiol (E2) levels (422 vs 576, *P* < 0.001), LH levels (4.03 vs 2.94, *P* < 0.001) on the day of triggering, and trigger-to-retrieval time (34.85 vs 35.05 h, *P* < 0.001). There was no significant difference in the use of double-lumen oocyte retrieval needles (92.31 vs 87.02%, *P* = 0.113) (Table 1).

**Table 1:** Patient demographics and baseline characteristics. The data are presented as the mean ± SD, *n* (%) or median (IQR).

Characteristics	Egg acquisition rate group		*P*-value
	<0.5	≥0.5	
Total *n*	113	2,481	
Age	36.4 ± 5.8	36.7 ± 5.7	0.565*
BMI	23.5 ± 3.7	23.5 ± 4.9	0.987*
Infertility duration	4.3 ± 3.8	4.1 ± 3.7	0.619*
Infertility type			0.071^†^
Primary infertility	43 (38.05%)	746 (30.07%)	
Secondary infertility	70 (61.95%)	1,735 (69.93%)	
Infertility factor			0.902^‡^
Tubal factor	62 (54.87%)	1,372 (55.30%)	
Endometriosis	10 (8.85%)	205 (8.26%)	
Ovulatory dysfunction	8 (7.08%)	222 (8.95%)	
Male factor infertility	4 (3.54%)	110 (4.43%)	
DOR	29 (25.66%)	572 (23.06%)	
bFSH	10.5 (7.5, 16.2)	10.3 (7.5, 12.4)	0.083^§^
bLH	5.30 (3.87, 7.19)	4.80 (3.51, 6.15)	0.026^§^
bPRL	15 (10, 17)	16 (11, 18)	0.429^§^
bE2	39 (24, 50)	40 (27, 53)	0.224^§^
bT	0.25 (0.13, 0.28)	0.21 (0.13, 0.28)	0.094^§^
bP	0.31 (0.16, 0.47)	0.31 (0.18, 0.46)	0.965^§^
AMH	0.48 ± 0.39	0.68 ± 0.57	<0.001*
AFC	2.93 ± 2.03	4.05 ± 2.08	<0.001*
COS protocol			<0.001^†^
Agonist protocol	8 (7.08%)	457 (18.42%)	
Antagonist protocol	60 (53.10%)	1,347 (54.29%)	
Natural cycle and microstimulation	21 (18.58%)	186 (7.50%)	
PPOS	24 (21.24%)	491 (19.79%)	
GH pretreatment	0 (0.00%)	13 (0.52%)	>0.999^‡^
Gn dosage	2,487 ± 1,362	2,406 ± 932	0.534*
Gn duration	8.97 ± 3.83	8.78 ± 2.91	0.605*
Gn starting dosage	300 (225, 300)	300 (225, 300)	0.952^§^
HCG day FSH	18.5 (16.6, 19.9)	18.5 (15.2, 21.0)	0.681^§^
HCG day E2	422 (221, 782)	576 (326, 951)	<0.001^§^
HCG day LH	4.03 (2.57, 6.18)	2.94 (1.68, 4.69)	<0.001^§^
HCG day P	0.35 (0.19, 0.64)	0.35 (0.21, 0.57)	0.951^§^
Number of large follicles on HCG day	2.53 ± 2.11	2.93 ± 1.94	0.050*
Endothelial thickness on HCG day	8.3 ± 3.1	8.7 ± 2.9	0.207*
Trigger protocol			0.656^‡^
HCG	106 (94.64%)	2,366 (95.63%)	
GnRHa	3 (2.68%)	47 (1.90%)	
HCG + GnRHa	3 (2.68%)	61 (2.47%)	
HCG dosage	9,687 ± 1,885	9,633 ± 1,872	0.765*
Trigger-to-retrieval time	34.85 (34.30, 35.28)	35.05 (34.77, 35.65)	<0.001^§^
Use of a double-lumen oocyte retrieval needle	96 (92.31%)	2,111 (87.02%)	0.113^†^

Statistical tests were performed using:

* Welch two sample *t*-test.

^†^ Pearson’s chi-squared test.

^‡^ Fisher’s exact test.

^§^ Wilcoxon rank sum test.

The high oocyte retrieval group had significantly greater numbers of total oocytes retrieved, metaphase II (MII) oocytes, two-pronuclei (2 PN) zygotes, normally cleaved embryos, and available embryos (all *P* < 0.001). No statistically significant differences were found in the blastocyst formation rate or the proportion of blastocyst transfers (*P* > 0.05). A significant difference was observed in the distribution of the number of transferred embryos (*P* = 0.039). Clinical pregnancy rate (41.67 vs 43.06%, *P* = 0.923), ectopic pregnancy rate, and miscarriage rate showed no significant differences. Although the live birth rate was higher in the high oocyte retrieval group, the difference was not statistically significant (*P* = 0.356). However, the cumulative pregnancy rate (CLPR) and cumulative live birth rate (CLBR) were significantly higher in the high oocyte retrieval group than in the LOR group (both *P* < 0.001) (Table 2).

**Table 2:** Patients laboratory index and clinical outcomes. The data are presented as *n* (%) or median (IQR).

Characteristics	Oocyte retrieval rate grouping		*P*-value
	<0.5	≥0.5	
Total *n*	113	2,481	
Total number of oocytes acquired	0.00 (0.00, 1.00)	3.00 (2.00, 5.00)	<0.001*
Total MII	0.00 (0.00, 1.00)	3.00 (1.00, 4.00)	<0.001*
Total 2 PN	0.00 (0.00, 1.00)	2.00 (1.00, 3.00)	<0.001*
Total number of normal oocytes	0.00 (0.00, 1.00)	2.00 (1.00, 3.00)	<0.001*
Number of embryos available	0.00 (0.00, 0.00)	1.00 (0.00, 2.00)	<0.001*
D3 good quality embryo	0.00 (0.00, 0.00)	0.00 (0.00, 1.00)	<0.001*
Blastocyst formation rate	0.00 (0.00, 0.50)	0.50 (0.17, 1.00)	0.110*
Number of blastocyst cultures	0.00 (0.00, 0.00)	0.00 (0.00, 0.00)	<0.001*
Number of blastocysts formed	0.00 (0.00, 0.00)	0.00 (0.00, 0.00)	<0.001*
Whether transfer blastocyst	0 (0.00%)	58 (6.00%)	>0.999^†^
Number of embryo transfer			0.039^‡^
1	9 (75.00%)	436 (45.13%)	
2	3 (25.00%)	530 (54.87%)	
Clinical pregnancy rate	5 (41.67%)	416 (43.06%)	0.923^‡^
Ectopic pregnancy rate	0 (0.00%)	9 (2.16%)	>0.999^†^
Miscarriage rate	3 (60.00%)	95 (22.84%)	0.085^†^
Live birth rate	2 (16.67%)	313 (32.40%)	0.356^†^
CLPR	8 (7.14%)	770 (34.51%)	<0.001^‡^
CLBR	4 (3.57%)	570 (25.97%)	<0.001^‡^

Statistical tests were performed using.:

* Wilcoxon rank sum test.

^†^ Fisher’s exact test.

^‡^ Pearson’s chi-squared test.

### Feature selection and parameter building

**Figure 1:**
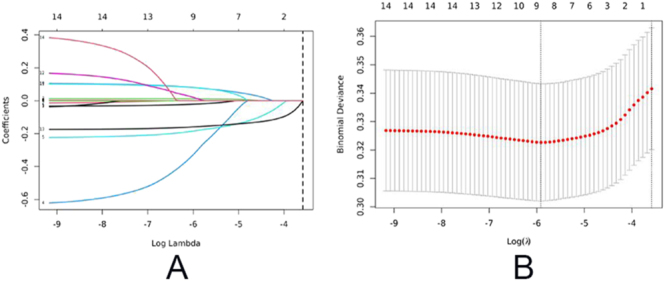
Variable selection using the least absolute shrinkage and selection operator (LASSO) regression algorithm. (A) LASSO regression path diagram. (B) LASSO coefficient profiles of the characteristics. Parameters were screened out by ten-fold cross-validation and using lambda.1se as the criteria.

Candidate predictor variables initially considered in this study included age, BMI, duration and type of infertility, infertility factors, basal sex hormone levels, AMH, AFC, total Gn dosage, Gn duration, Gn starting dosage, COS Protocol, hormone levels on HCG day, number of dominant follicles on HCG day, type of trigger protocol, HCG dosage, trigger-to-retrieval time, and use of a double-lumen oocyte retrieval needle. LASSO regression analysis was applied to the entire cohort, resulting in the shrinkage of most variable coefficients to zero and retention of only a subset of predictive features. The final model included an intercept (3.275) and the following non-zero coefficient variables: age category (−0.020), basal FSH (0.011), AMH (−0.302), AFC (−0.181), LH level on the HCG day (0.078), P level on HCG day (0.079), number of large follicles on HCG day (0.013), and trigger-to-retrieval time (−0.158). Variables with coefficients equal to zero – such as BMI, total gonadotropin dosage and duration, FSH and E2 levels on HCG day, and use of a double-lumen needle – were considered to have limited predictive value. The LASSO regression path plots and coefficient distributions are presented in Fig. 1A and B. This model highlights the advantages of LASSO in variable selection and model simplification. Subsequently, multivariate logistic regression was performed across the entire cohort, as shown in Table 3.

**Table 3:** Results of multivariate logistic regression.

Characteristics	*n*	Event *n*	OR	95% CI	*P*-value
Age	2,594	113	0.97	0.93, 1.00	0.038*
FSH	2,594	113	1.02	0.99, 1.04	0.189
E2	2,594	113	1	1.00, 1.01	0.018*
P	2,594	113	1.28	1.16, 1.65	<0.001*
AMH	2,594	113	0.5	0.26, 0.88	0.023*
AFC	2,594	113	0.81	0.72, 0.91	<0.001*
Number of large follicles on HCG day	2,594	113	1.09	0.96, 1.23	0.177
HCG day LH	2,594	113	1.08	1.00, 1.18	0.052
HCG day P	2,594	113	1.1	0.97, 1.20	0.044*
Trigger-to-retrieval time	2,594	113	0.81	0.74, 0.87	<0.001*

OR, odds ratio; CI, confidence interval.

* *P* < 0.05.

### Development of an individualized prediction model

**Figure 2:**
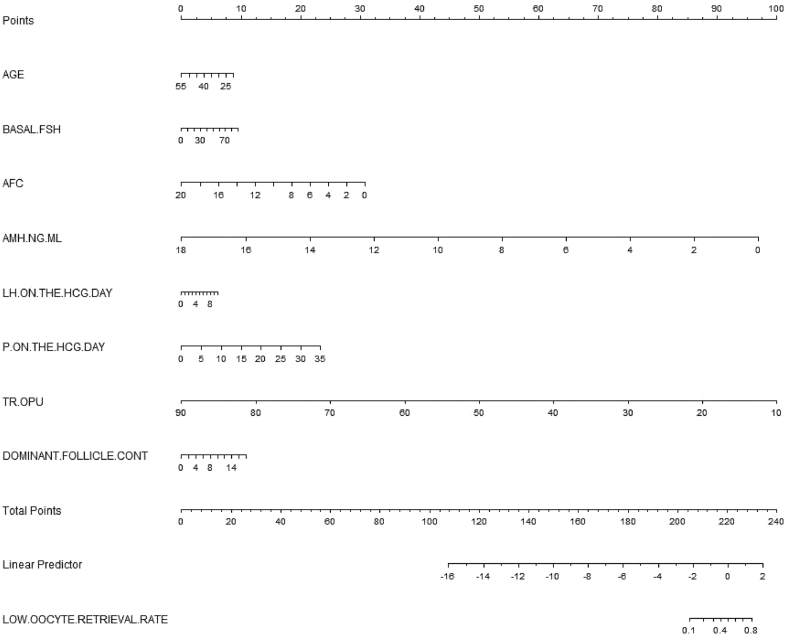
Nomogram to predict the risk of LOR rate. The points for each variable were determined by drawing a vertical line from the value of the variable to the point scale. The points for each variable were determined by drawing a vertical line from the value of the variable to the point scale. The total points were calculated by summing the individual points, and a downward line was drawn from the ‘total points’ axis to intersect with the ‘probability of LOR’ axis. The total points were calculated by summing the individual points, and a downward line was drawn from the ‘total points’ axis to intersect with the ‘probability of LOR rate’ axis, which provided the estimated probability.

The developed model for estimating the risk of LOR rate used the selected variables, including eight independent predictors (age, basal FSH, AFC, AMH, LH on the HCG day, P on the HCG day, TR-OPU, and dominant follicle count) as indicators. The nomogram for prediction is depicted in Fig. 2. Each parameter was assigned a vertical extension (shown in the top points bar) individually. The total score was determined by summing up the scales for each factor. The overall point projected on the bottom scale suggests the likelihood of LOR rate.

### Validation of the nomogram

**Figure 3:**
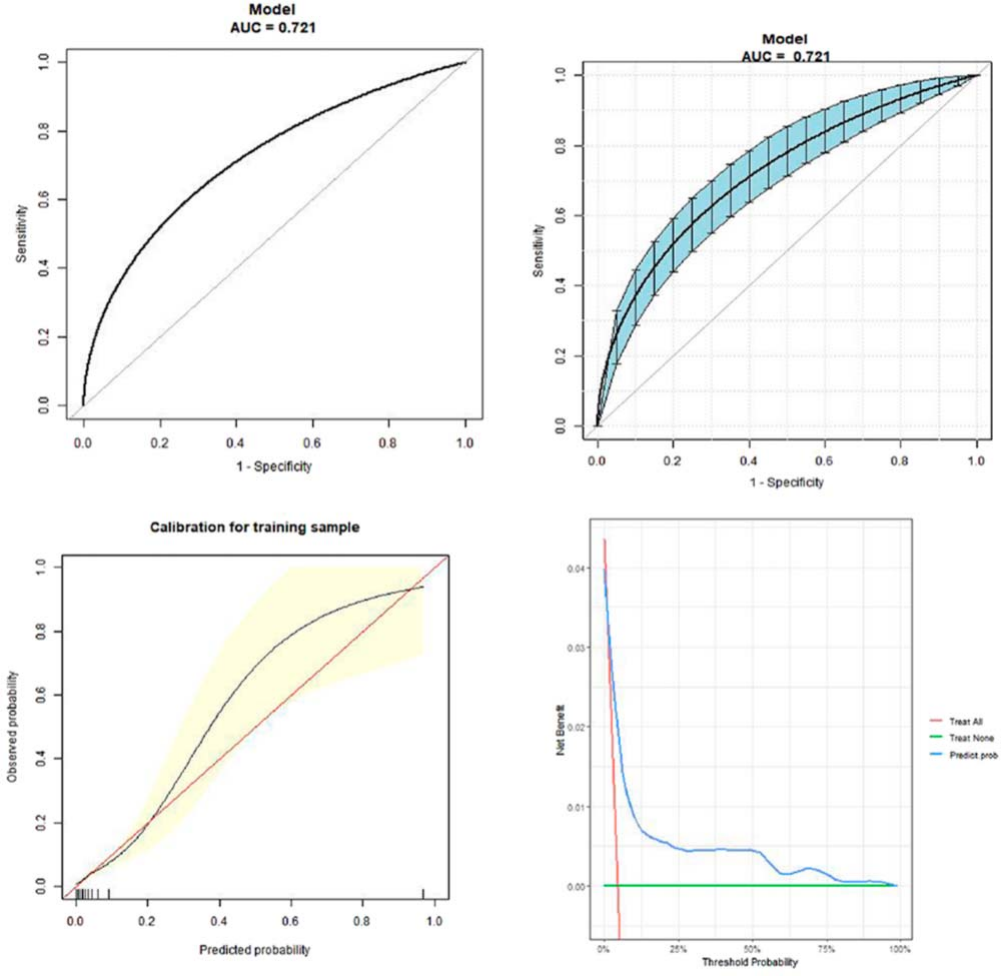
Model evaluation and validation results. The AUC confidence intervals and significance testing were derived through the bootstrap approach with 500 resampling iterations (A and B). Calibration curves for the nomogram are shown in (C). (D) DCA curves associated with the nomogram.

To assess the predictive performance of the model, receiver operating characteristic (ROC) curve analysis was performed. The results demonstrated good discriminative ability, with an area under the curve (AUC) of 0.721 (95% CI: 0.678–0.772). The AUC confidence intervals and significance testing were derived using the bootstrap method with 1,000 resamples (Fig. 3A and B). The corrected Harrell’s C-index was 0.695, indicating good internal validation. Figure 3C presents the calibration plot for the nomogram model, indicating strong concordance between the predicted and observed rates of LOR. The calibration curve closely aligns with the ideal reference line, suggesting that the model’s predictions are consistent with the actual outcomes. Figure 3D shows the DCA for the nomogram, highlighting its clinical net benefit. Higher risk threshold probabilities are associated with greater potential predictive error in clinical application, emphasizing the need for cautious interpretation. In addition, when comparing the predictive accuracy of individual variables to that of the combined model using ROC curves, the nomogram exhibited significantly higher AUC values (Table 4).

**Table 4:** Predictive accuracy of the model and various variables for LOR rate.

Test	AUC	95% CI	BT	SP	SN	PPV	NPV
Age	0.5034	0.4444–0.5593	36.5	0.5312	0.5575	0.0514	0.9635
Basal FSH	0.548	0.4927–0.6087	16.19	0.8863	0.2566	0.0932	0.9632
AFC	0.6538	0.5905–0.703	3.5	0.6102	0.6549	0.0711	0.9749
AMH.NG.ML	0.6399	0.5852–0.6891	0.225	0.8662	0.3717	0.1123	0.968
LH on HCG day	0.6112	0.5615–0.6652	2.545	0.4329	0.7699	0.0582	0.9764
P on HCG day	0.4901	0.4334–0.5488	0.1645	0.8428	0.2301	0.0625	0.9601
TR-OPU	0.627	0.5783–0.691	34.875	0.6622	0.531	0.0668	0.9688
Dominant follicle count	0.5887	0.5235–0.6709	1.5	0.7485	0.5398	0.0891	0.9728
Model	0.7212	0.6783–0.7717	−2.9889	0.7243	0.646	0.0964	0.9782

LOR, low oocyte retrieval; BT, best threshold; SP, specificity; SN, sensitivity; PPV, positive predictive value; NPV, negative predictive value.

## Discussion

### Research overview and core innovations

This study developed and validated a multivariable prediction model for a LOR rate in patients with DOR. By focusing on retrieval efficiency – the ratio of oocytes retrieved to mature follicles – rather than the absolute oocyte count, our model addresses a pivotal clinical question for this challenging population: ‘given the follicles we have grown, how efficiently can we harvest them?’ A low retrieval rate signifies a disproportionate loss of potential, pointing to issues potentially related to final oocyte maturation, trigger efficacy, or technical aspects of the retrieval. The nomogram is designed for deployment immediately after the HCG trigger, providing a quantitative risk estimate to manage patient expectations and prompt an insightful review of the completed stimulation cycle.

### Interpretation of predictors

The predictors retained in our final model, selected through the LASSO regression, highlight the multifactorial nature of LOR. As expected, established markers of ovarian reserve, such as AMH and AFC, were strongly associated with the outcome. It is noteworthy that the number of large follicles on the HCG day was also retained by the LASSO algorithm. Although its individual *P*-value in the subsequent logistic regression was not statistically significant (*P* = 0.177), its selection through this data-driven process indicates that it provides valuable information for risk stratification within the context of the full model. This is biologically and clinically coherent, as this variable represents the fundamental pool of available oocytes.

A nuanced interpretation is required for the trigger-to-oocyte retrieval (TR-OPU) interval. Our model identified a statistically significant but clinically modest association between TR-OPU time and LOR risk. The effect magnitude in our model is distinct in both scale and implication from the large, intentional interval extensions (>36 h) investigated in other studies to improve oocyte maturity (Wang *et al.* 2011). The biological plausibility of TR-OPU timing influencing outcomes, potentially through mechanisms involving follicular microenvironment maturation (Vrtačnik-Bokal *et al.* 2004, Alhilali *et al.* 2019, Babayev & Duncan 2022), supports its inclusion as a predictive feature, but clinicians should not extrapolate our finding to justify altering standard protocols. This hypothesis-generating finding justifies more focused investigation into the precise ‘timing window’ for different patient profiles. Future studies, perhaps measuring biomarkers of follicular maturity, are needed to move from our predictive signal to a causal understanding that could inform personalized trigger protocols.

### Model performance, clinical application and precautions

The model demonstrated moderate yet acceptable discrimination, with an area under the curve (AUC) of 0.721. Calibration curves indicated an overall good fit, and DCA suggested favorable clinical utility across a range of reasonable risk thresholds.

A critical consideration is the fundamental distinction between prediction and causation. This model estimates the risk of LOR under the specific treatment practices observed in our cohort, aligning with a prognostic ‘ignore treatment’ predictimand framework (van Geloven *et al.* 2020). Consequently, the coefficients for treatment-related variables (e.g., total gonadotropin dose) must not be interpreted as causal effects. The model’s utility is prognostic and decision-supportive; it is not intended to guide specific manipulations of treatment variables during the ongoing cycle.

The primary value of our model lies in its predefined deployment point – after the HCG trigger and before oocyte retrieval – when all predictor values are known. At this juncture, it serves two key functions: i) to manage patient expectations by providing a transparent, individualized risk estimate for the imminent procedure, thereby mitigating psychological distress, and ii) to flag a high-risk case, prompting the clinical team to conduct a thorough, retrospective analysis of the completed cycle. This analysis can generate valuable hypotheses for protocol optimization in subsequent treatment attempts.

The strengths of our study include a large, well-defined cohort of first-time IVF patients with DOR and a robust model-building process utilizing LASSO and internal validation. Several limitations must be acknowledged. Its single-center, retrospective nature may introduce selection bias and limits generalizability, necessitating external validation. Furthermore, the relatively low event rate for LOR, while statistically manageable, may affect model stability. Finally, we were unable to account for all potential confounders, such as detailed genetic profiles.

## Conclusion

In conclusion, we have developed and internally validated a multifactorial prediction model for LOR rate in patients with DOR. This nomogram provides a promising tool for exploratory use, which could aid in personalized patient counseling and inform clinical decision-making by stratifying LOR risk. Nevertheless, its definitive role as a clinical decision-support tool requires rigorous external validation across diverse populations before any broader clinical implementation.

## Declaration of interest

The authors declare that there is no conflict of interest that could be perceived as prejudicing the impartiality of the work reported.

## Funding

This work was supported by the Henan Province Medical Science and Technology Research Plan (Grant Number LHGJ20220049).

## Author contribution statement

XW supervised the whole research, including the processes, conception, design, and completion. She also contributed to the analysis of the study results and made amendments to the publication. JL made significant contributions to the analysis of the data and also wrote the first version of the paper. C-YD conducted data collecting and authored portions of the text. LW, P-ZT and C-LZ made substantial contributions to design of the work and revised it. Each author made contributions to the paper and endorsed the submitted version.

## Data availability

The datasets used and/or analyzed during the current study are available from the corresponding author on reasonable request.
